# Body mass index and health related quality of life in elementary school children: a pilot study

**DOI:** 10.1186/1477-7525-6-77

**Published:** 2008-10-09

**Authors:** Lei Zhang, Peter J Fos, William D Johnson, Vafa Kamali, Reagan G Cox, Miguel A Zuniga, Theresa Kittle

**Affiliations:** 1 Mississippi State Department of Health, Jackson, MS, USA; 2 University of Texas at Tyler, Texas, USA; 3 Louisiana State University, Baton Rouge, LA, USA; 4 University of Southern Mississippi, Hattiesburg, Mississippi, USA; 5 Vanderbilt University, Nashville, TN, USA; 6 Texas A&M Health Science Center, South Texas, McAllen, TX 78503, USA

## Abstract

**Background:**

We investigated the relationship between Body Mass Index (BMI) and health-related quality of life (HRQOL) indicated by baseline health status in elementary school children.

**Methods:**

Data were obtained via parents whose children enrolled in an elementary school, kindergarten to fourth grade, in southern Mississippi in spring 2004. Parents completed the *SF-10 for Children™*, a brief 10-item questionnaire designed to measure children's HRQOL on a voluntary basis.

**Results:**

A total of 279 parents completed the questionnaires for their children. On average, physical and psychosocial summary scores, major indicators for HRQOL, were significantly higher among the elementary school children in our study relative to those from U.S. children overall (p < 0.0001 and p = 0.0007, respectively). Males tended to have better physical functioning than their female classmates, whereas females had better psychosocial health. Overall, except for third graders, the physical summary scores increased as grade level increased. The means for psychosocial score fluctuated without a clear pattern over the five grade levels. High level of BMI was significantly associated with children's physical summary scores below 50, a norm used for U.S. children (p = 0.003). Gender and grade were not significant predictors of children's physical and psychosocial scores.

**Discussion:**

This study can be used as baseline information to track changes over time, in BMI and health status among the elementary school children. In addition, this study can be used to investigate relationships between BMI, health status, intellectual ability, and performance in school.

**Conclusion:**

The findings suggest that programs designed to encourage children to lose weight in a healthy manner, thus reducing their BMI, could improve the physical and psychosocial health, and subsequently increase HRQOL.

## Background

Health is a concept that is broader than simply the absence of disease, but encompasses physical, social, mental, and emotional well-being. Health-related quality of life (HRQOL) is a notion that attempts to merge all aspects of overall quality of life related to general health. These life circumstances have been identified as both physical and mental [1]. Indices of HRQOL represent physical and mental perceptions, and health risks, functional status, and socioeconomic status. At the population level HRQOL measures conditions and resources that affect the perceptions of health and functional status. In this context, HRQOL can be seen as an expansion to the concept of health which then allows for encompassing the physical and mental needs in a population [2].

HRQOL is becoming a popular measurable outcome that questions the perceived physical and mental health and function, and is generally considered an appropriate and adequate measure of health care service needs and intervention outcomes [3]. HRQOL measurements allow for scientific demonstration of the impact of quality of life on health. HRQOL is related to self-reported characteristics associated with chronic diseases and risk factors. HRQOL surveillance can provide insights to the identification of sub-groups in a population who have perceived poor health, and then provide guidelines for targeting of high priority interventions.

Several measures have been used to assess HRQOL and related functional status, and to describe these notions in the context of health status. These measures include: Medical Outcomes Study Short Forms (SF-36, SF-12, *SF-10 for Children™*, and SF-8), the Sick Impact Profile, and Coop Charts. The SF-survey series are used by the Center for Medicare and Medicaid Services and the National Committee for Quality Assurance's Health Plan Employer Data Information Set (HEDIS 3.0) to evaluate the quality of care that is provided in managed care plans and other health care facilities [4].

Health status is determined by several factors, including physical health and functional status, and its measurement involves these dimensions and associated objective and subjective measures. Health status measurement is accomplished as either a health status index or profile. An index is characterized by a single score representing health status. On the other hand, a health status profile provides a multidimensional evaluation of all aspects of health. Health profiles are popular in situations where the interaction of the physical, social, mental and emotional determinants of health are of interest. Health indices are useful in health policy and economic evaluation, because a single score is useful in making choices and decisions. The SF-series and the *SF-10 for Children™ *are examples of a health profile.

Body Mass Index (BMI) is a tool for indicating a person's weight status. It is a measure of body weight for a specified height. BMI correlates with body fat and a high level of body fat may increase the risk of developing diseases. The relation between fatness and BMI differs with age and gender. As BMI increases, the risk for some disease increases. In adults, BMI is often divided into the following categories with respect to height: (1) underweight, (2) normal weight, (3) overweight, and (4) obese. Common conditions that are related to being overweight or obese include: premature death, cardiovascular disease, high blood pressure, osteoarthritis, some cancers, and diabetes. However, BMI is only one of many factors used to predict risk for disease. Different from adults, BMI for children is frequently categorized as (1) underweight, (2) normal weight, (3) at risk for overweight, and (4) overweight. Children's body fatness changes over the years as they grow. Also, girls and boys differ in their body fatness as they mature, so the BMI for children, also referred to as BMI-for-age, is a gender and age specific measurement [5,6]. In 2007, American Medical Association Expert Committee on the Assessment, Prevention, and Treatment of Child and Adolescent Overweight and Obesity recommended to use "overweight" and "obese" to replace terminology "at risk for overweight" and "overweight" respectively. Since the study was conducted prior to the recommendation, the traditional classification on BMI was used in this study so the findings are consistent with the previous records.

Based on the 2007 data from the National Behavioral Risk Factor Surveillance System and Youth Risk Behavior Surveillance System, Mississippi led the nation in obesity among adults and public high school students. Similar population data are not available for Mississippi elementary school children. The main objective of this study is to investigate if BMI is associated with HRQOL in a small sample among elementary school students to gain insight about correlates of early onset of obesity.

## Methods

The *SF-10 for Children™ *is a brief 10-item questionnaire designed to measure children's HRQOL. Study questionnaires were composed of standardized *SF-10 For Children™ *questions, as well as questions requesting demographic characteristics (age, gender and grade level) and personal characteristics (height and weight). A detailed description of the survey is reported elsewhere [7]. In the spring of 2004, our investigation surveyed parents of children enrolled in an elementary school, kindergarten to fourth grade, in southern Mississippi. Parents completed the *SF-10 for Children™ *on a voluntary basis.

Physical (PHS-10) and psychosocial (PSS-10) summary scores were calculated according to *SF-10 for Children™ *survey guidelines [7]. Higher scores indicate more favorable physical and psychosocial functioning. Five survey questions are required for the physical score and five are necessary for the psychosocial score. Summary score were not calculated for students with missing responses to required questions.

BMI was calculated for children with reported height and weight data. Insufficient height and/or weight measurements were reported for 88 (31.5%) of sample participants. Height measurements were rounded to the nearest inch and weight measurements were rounded to the nearest pound. BMI was calculated as weight in pounds divided by height in inches, squared and multiplied by 703 to obtain the standard BMI measurement unit of (kilograms/meters^2^). Data from the 2000 Centers for Disease Control and Prevention (CDC) gender-specific, BMI-for-age growth charts were used to determine BMI percentiles for individuals [8]. Children were classified into four categories: (1) underweight (BMI is less than or equal to 5th percentile), (2) normal weight (BMI is greater than 5th- but less than 85th percentile), (3) at risk for being overweight (BMI is greater than or equal to 85th- but less than 95th percentile) and (4) overweight (BMI is greater than or equal to 95th percentile). Of the children with sufficient data to calculate BMI, 12 (6.3%) were classified as "underweight" and were excluded from analyses.

SAS 9.1 (SAS Institute Inc., Cary, NC) was used for all statistical calculations. Summary statistics (mean, standard deviation and range) were calculated for PHS-10 and PSS-10 scores. Mean PHS-10 and PSS-10 scores were calculated for the total sample and also for gender and grade level sub-groups. A score of 50 was established as the norm for U.S. children for both physical and psychosocial health. The one-sample t-test was used to determine if the physical and psychosocial mean scores differed from the national averages. PHS-10 and PSS-10 scores each were categorized into three groups: "substantial impact" (score < 47), "some impact" (score 47–50), and "little or no impact" (scores ≥ 50). The prevalence of children in each score category was calculated based upon their BMI classification. Physical and psychosocial scores were regrouped into dichotomous variables where students with scores less than 50 were considered to have poor physical or psychosocial functioning. Logistic regression was used to investigate factors that may be associated with poor functioning. Effects of the following correlated factors were examined: gender, grade level and BMI.

## Results

The sample included 279 students from a single elementary school in Mississippi. Slightly more females than males were included in the sample (53% vs. 47%). Children represented five consecutive grade levels, from kindergarten through fourth grade. The percentage of students in each respective grade level (K, 1st, 2nd, 3rd, and 4th) was 20%, 21%, 25%, 18% and 16%.

**Figure 1:**
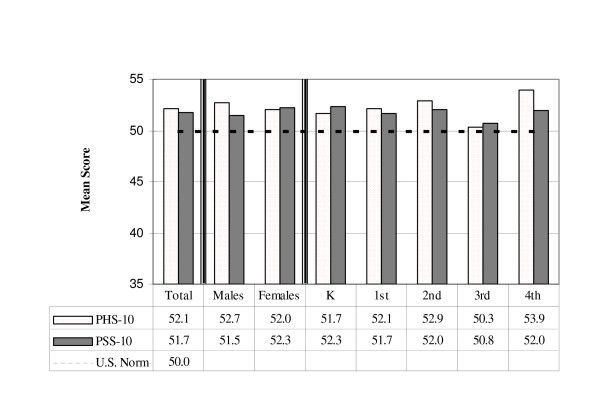
Mean PHS-10 and PSS-10 scores by gender and grade level.

Figure 1 contains comparisons of mean physical and psychosocial summary scores for the sample population. On average, children in the sample had significantly higher physical summary scores than children in the general U.S. population (p < 0.0001). Children's psychosocial summary scores were also significantly higher than the U.S. norm (p = 0.0007). Male students tended to have better physical functioning than their female classmates, whereas female students had better psychosocial functioning. Overall, physical summary scores increased as grade level increased except for those from the third grade. Mean psychosocial scores fluctuated without a clear pattern over the five grade levels.

Physical summary scores were calculated for 264 students. The mean physical summary score was 52.1 with range from 6.3 to 56.7. Table 1 depicts the frequency of students with physical summary scores in each category based upon their BMI classification. The percentage of children reporting physical summary scores greater than or equal to 50 decreased as BMI increased. Likewise, as BMI increased the percentage of children reporting physical scores between 47 and 50 decreased. Although the percentage of children reporting PHS-10 scores below 47 decreased with increasing BMI, there was no linear trend.

**Table 1:** PHS-10 scores by BMI category

	**PHS-10 Score Category**		
	**< 47**	**47–50**	**≥ 50**
BMI Category	Frequency (Percent)	Frequency (Percent)	Frequency (Percent)

Normal weight	9 (39.1)	10 (55.6)	88 (58.7)
At risk for overweight	5 (21.7)	4 (22.2)	27 (18.0)
Overweight	8 (34.8)	4 (22.2)	24 (16.0)

§Students with BMI classification of "underweight" were excluded due to small frequencies. Therefore, column percentages do not necessarily total to 100%.

Psychosocial summary scores were available for 274 students. The mean psychosocial score was 51.7 with range from 13.9 to 60.8. Psychosocial scores were grouped into categories based upon *SF-10 for Children™ *survey guidelines. Table 2 shows the frequency of students with psychosocial summary scores in each category based upon their BMI classification. As BMI increased the percentage of children reporting psychosocial summary scores greater than or equal to 50 decreased. Similarly, the percentage of students reporting the poorest psychosocial health (PSS-10 scores < 47) decreased as BMI increased. The percentage of children reporting physical scores between 47 and 50 decreased as BMI increased, however the trend was not linear.

**Table 2:** PSS-10 scores by BMI category

	**PSS-10 Score Category**		
	**< 47**	**47–50**	≥ **50**
BMI Category	Frequency (Percent)	Frequency (Percent)	Frequency (Percent)

Normal weight	15 (44.1)	8 (47.1)	84 (60.0)
At risk for overweight	10 (29.4)	2 (11.8)	24 (17.1)
Overweight	7 (20.6)	6 (35.3)	23 (16.4)

§Students with BMI classification of "underweight" were excluded due to small frequencies. Therefore, column percentages do not necessarily total to 100%.

The majority of children in this sample scored 50 or higher on the physical and psychosocial summary score scales. Only 41 (21.5%) children reported a physical summary score less than 50. Of these children, 36 also had data available for gender, grade and BMI. The model for physical summary score was significant (p = 0.026). Increasing BMI was significantly associated with physical summary scores below 50 (p = 0.003). Gender and grade level were not significantly correlated with PHS-10 scores.

A total of 51 (26.7%) children reported a psychosocial score less than 50. Of these, 48 had data available for the selected independent factors. The model for psychosocial summary score was not significant (p = 0.193).

## Discussion

The general health of youth in Mississippi concerns parents and community leaders. It is encouraging to see that young children in this Mississippi school currently enjoy physical and psychosocial functioning at a level higher than in the general U.S. population. In order to continuously monitor children's health status, however, similar but more inclusive studies are necessary as researchers need to further investigate factors that could potentially impede the health of our children.

Of the 279 children for which physical and psychosocial summary scores were calculated, 73% had PHS-10 scores ≥ 50 and 69% had PSS-10 scores ≥ 50. Although the majority of students apparently enjoy very good HRQOL, the prevalence of high scores may be distorted by sample characteristics. The children who attend this elementary school are predominantly white and from middle- to upper class families. Thus, the demographic profile of the sample is not representative of either the entire state of Mississippi or the general U.S. population. We hypothesize that a sample with a higher percentage of minority children and with diverse family income levels would most likely produce a lower prevalence of children with high physical and psychosocial scores.

Our results are reasonably consistent with previous research. A few studies have identified high BMI or obesity as a significant factor associated with poor HRQOL [9-11]. Investigators have consistently reported high BMI to be significantly associated with lower physical health scores. The association appears to hold for a variety of youth populations: a community-based sample of younger adolescents (ages 8–11) [9], a nationally representative sample of older adolescents (ages 12–20) [10], and a sample of severely obese children and adolescents (ages 5–18) [11]. Previous studies have not consistently reported a significant association between BMI and psychosocial health among children [9-11]. Overweight children scored significantly lower on the psychosocial health score compared to children of normal weight in a Cleveland, Ohio community-based sample [9]. Children included in the sample for the Cleveland study were not representative of Mississippi children. Specifically, the sample population was slightly older (ages 8–11) and 31.5% were of minority racial descent. Furthermore, the definition for normal weight (BMI is greater than or equal to 20th- but less than 85th percentile) was more exclusive than our definition and the logistic regression model utilized this category for comparison purposes. Differences in methodology, sample size and sample characteristics may explain our findings that suggest psychosocial health is not significantly associated with a child's BMI.

Our findings suggest that males enjoy a higher quality of physical health compared to their female classmates. Conversely, females score higher on the psychosocial functioning scale.

Our study has several limitations. The study participants were not randomly selected from some larger population. Parents were given the option to answer or decline to answer the study questionnaire for their children. No demographic information was collected for the students whose parents declined participation in the study, so we were unable to examine whether these students differed from the students who participated. Further, a larger sample may have enhanced our ability to detect statistically significant relationships.

The instrument used to collect survey data relied upon parental evaluation of child health. While proxy reporting of health indicators by parents/guardians is generally valid for children in this age range, data may be distorted by factors that are not controllable, e.g. parent-child relationships or parental perception of child functioning.

Height and weight were collected via parental report. The accuracy of measurements was not confirmed. Moreover, over 30 percent of respondents did not report height and weight. This limited our ability to evaluate the relationship between BMI and physical and psychosocial health. Despite these limitations, our study has shown that parent-reported health status of children was successfully collected using the *SF-10 for Children™ *survey instrument. Also, baseline BMI and health status data were determined for a population of elementary school children in a state with the highest prevalence of adult obesity. This baseline information can be used to track changes over time, in BMI and health status. In addition, this study can be used as baseline information to investigate relationships between BMI, health status, intellectual ability, and performance in school.

## Conclusion

This study provides evidence of an association between BMI and HRQOL in elementary school children. The impact of being overweight appears to affect both the physical and psychosocial health of children as young as 5 years of age. In fact, our findings suggest that the health of younger children may be more disturbed by their high BMI, compared to children a couple years older. While our findings do not suggest causality, the association between childhood obesity and childhood HRQOL is something that should not be overlooked. Many researchers are concerned with childhood eating and exercise habits that may contribute to poor health as adults. Our findings suggest that a child's present health may be less than optimal due in part to their elevated BMI. Further research is necessary to elucidate other aspects of the relationship between BMI and children's HRQOL. However, the findings from this study suggest that programs designed to encourage children to lose weight in a healthy manner, thus reducing their BMI, could also improve their physical and psychosocial health.

## Competing interests

The authors declare that they have no competing interests.

## Authors' contributions

LZ led data analysis and writing, and supervised all aspects of its implementation. PJF originated the study and contributed writing. WDJ contributed interpreting results and writing. VK proposed the ideas and posed a number of substantive questions related to data collection and analysis. RGC contributed data analysis and writing. MAZ provided guidance on using *SF-10 for Children *^TM^ related to data collection and analysis. TK managed the database and did data entry. All authors read and approved the final manuscript.
